# Validation of Folate-Enriched Eggs as a Functional Food for Improving Folate Intake in Consumers

**DOI:** 10.3390/nu8120777

**Published:** 2016-11-30

**Authors:** Leslie Altic, Helene McNulty, Leane Hoey, Liadhan McAnena, Kristina Pentieva

**Affiliations:** Northern Ireland Centre for Food and Health, School of Biomedical Sciences, University of Ulster, Cromore Road, Coleraine BT52 1SA, UK; leslie.altic@ntlworld.com (L.A.); h.mcnulty@ulster.ac.uk (H.M.); l.hoey@ulster.ac.uk (L.H.); l.mcanena@ulster.ac.uk (L.M.)

**Keywords:** folate-enriched eggs, folate stability, food folate analysis, novel foods, functional foods

## Abstract

Functional foods enriched with folate may be beneficial as a means of optimizing folate status in consumers. We recently developed novel eggs enriched with folate through folic acid supplementation of the hen’s feed, but their potential to influence consumer folate status is unknown because the natural folate forms incorporated into the eggs may not necessarily be retained during storage and cooking. This study aimed to determine the stability of natural folates in folate-enriched eggs under typical conditions of storage and cooking. Total folate was determined by microbiological assay following tri-enzyme treatment in folate-enriched eggs and un-enriched (barn and free-range) on the day they were laid, after storage (up to 27 days) and after using four typical cooking methods (boiling, poaching, frying, scrambling) for different durations. On the day of laying, the folate content of enriched eggs was found to be significantly higher than that of un-enriched barn or free-range eggs (mean ± SD; 123.2 ± 12.4 vs. 41.2 ± 2.8 vs. 65.6 ± 18.5 µg/100 g; *p* < 0.001). Storage at refrigerator and room temperature for periods up to the Best Before date resulted in no significant losses to the folate content of folate-enriched eggs. Furthermore, folate in enriched eggs remained stable when cooked by four typical methods for periods up to the maximum cooking time (e.g., 135 ± 22.5, 133.9 ± 23.0 and 132.5 ± 35.1; *p* = 0.73, for raw, scrambled for 50 s and scrambled for 2 min, respectively). Thus, natural folates in folate-enriched eggs remain highly stable with little or no losses following storage and cooking. These findings are important because they demonstrate the feasibility of introducing folate-enriched eggs into the diet of consumers as functional foods with enriched folate content. Further studies will confirm their effectiveness in optimizing the biomarker folate status of consumers.

## 1. Introduction

The B-vitamin folate plays an established protective role in the prevention of neural tube defects (NTD) [1,2]. This discovery led to the introduction of worldwide recommendations for women planning a pregnancy to take folic acid (FA) supplements from before conception and up to the 12th gestational week in order to ensure optimal maternal status during the period of neural tube closure in early pregnancy. In more recent years, however, evidence is emerging to suggest that the achievement of optimal folate status may be important not only for women of child-bearing age, but for the general population. This is due to the increased recognition of the potential protective role of folate in the primary prevention of cardiovascular disease, particularly stroke [3,4], age-related cognitive impairment [5,6] and osteoporosis [7,8].

An evaluation of the current folate recommendations for women planning a pregnancy (i.e., based on the use of folic acid supplements) has shown that compliance is low [9,10] and consequently these recommendations have been ineffective in decreasing the incidence of NTDs in all European countries examined [11]. However, other countries, such as the US and Canada, have addressed this issue by introducing policies requiring mandatory FA fortification of cereal-grain products. This measure has resulted in a substantial increase in folate status of all populations in which such a policy has been introduced [12,13], a subsequent decrease in the incidence of NTDs [14,15] and possibly a reduction in stroke-related deaths [16]. Despite this evidence, many European governments have decided against the introduction of a mandatory FA fortification policy. This is primarily a result of safety concerns regarding chronic exposure of the general population to FA, the synthetic form of the vitamin, which, when consumed in high doses can lead to the appearance of unmetabolized FA in the circulation [17,18]. Previously, the most widely publicised risk of excess FA intake concerned the potential to mask the macrocytic anaemia of vitamin B_12_ deficiency, common in older adults, while allowing the associated irreversible neurological symptoms to progress [19]. However, other safety concerns have also been raised, most notably evidence suggesting the potential of high-dose FA to promote the growth of new or already existing but undiagnosed colorectal adenomas in those with pre-existing lesions [20].

With the lack of success of FA supplementation and concerns regarding FA-fortified foods, alternative approaches to achieving optimal folate status need to be considered. One option is the development of novel functional foods based on animal products enriched with natural forms of folate through the addition of high-dose FA to animal feed. Research has shown that eggs can be successfully enriched with folate by supplementing hens’ diets with FA, achieving a maximal folate content of about 2.5 times that of a normal egg [21,22,23], with a minimal (less than 10%) amount of unmetabolized FA [22]. 

Although our previous studies demonstrate that eggs can be successfully enriched with natural folates through folic acid supplementation of the hen’s feed, the potential of these eggs to influence consumer folate status is unknown because the natural folate forms incorporated into the eggs may not necessarily be retained during storage and cooking. As reduced forms of the vitamin, natural folates are labile and prone to oxidation, thus potentially leading to poor stability during processing and/or cooking [24,25,26]. The aim of the present study, therefore, was to investigate the effect of both storage and cooking on the stability of natural folates in folate-enriched eggs.

## 2. Materials and Methods 

### 2.1. Study Design

All eggs used in this study were produced by Skea Eggs (Donaghmore, Northern Ireland) and Devenish Nutrition (Belfast, Northern Ireland) according to the protocol for folate enhancement as previously described [20]. Each batch of eggs was collected from Skea Eggs on the day that they were laid (i.e., day of collection = day 1 for subsequent experiments). All storage and cooking experiments were carried out at the Northern Ireland Centre for Food and Health (Ulster University).

#### 2.1.1. Stability of Folates during Storage

**Figure 1:**
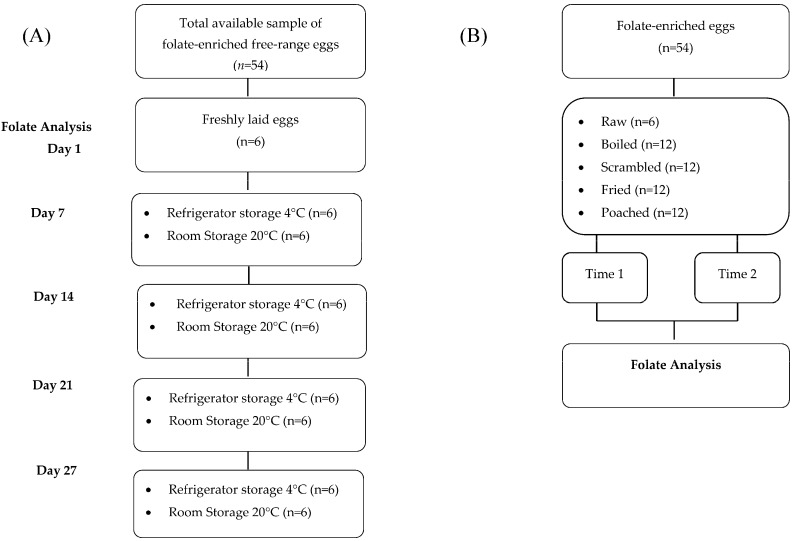
Flowchart of the experiments for the effect of storage (**A**) and cooking (**B**) on the stability of folate in folate-enriched eggs. Time 1 and time 2 refer to sampling times and correspond with the ‘soft’ and ‘hard’ consistency of cooked egg.

Three types of egg were collected: un-enriched (barn and free-range; *n* = 18 for each type) and folate-enriched (free-range; *n* = 54). Each group of eggs was divided into two sets; set 1 was stored at refrigerator temperature (4–7 °C) and set 2 was stored at room temperature (18–20 °C) for a total of 27 days (the maximum time allowed by Skea Eggs for the ‘Best Before’ date). On days 1, 7, 14, 21 and 27, samples of enriched eggs (*n* = 6) from each storage condition were weighed without the shell and then analyzed for folate content (Figure 1). Un-enriched eggs (barn and free-range) from each storage condition (*n* = 6) were weighed and analyzed for folate content only on days 1 and 27.

#### 2.1.2. Stability of Folates during Cooking

Cooking stability experiments were performed on two separate occasions using two separate batches of folate-enriched eggs. On each cooking occasion, a batch of 54 folate-enriched eggs was collected (Figure 1). Eggs were cooked by four typical cooking methods: boiling, poaching, frying, and scrambling, and the folate content was analyzed (*n* = 6) at two time points, to correspond to the ‘soft’ and ‘hard’ cooking of eggs by each cooking method (details below). Preliminary experiments were carried out for each type of cooking method to determine the cooking time (min) required to achieve the corresponding ‘soft’ and ‘hard’ consistencies of eggs. Samples of raw egg were also assessed for folate content (*n* = 6) at the time of each cooking experiment.

Prior to cooking, eggs for frying, poaching and scrambling were weighed without the shell. They were weighed again following cooking in order to take into account changes in weight due to water loss as a result of cooking. Eggs for boiling were weighed without the shell after cooking. For frying, poaching and scrambling experiments, all eggs were cooked individually whereas for boiling experiments the eggs were cooked together. *Boiling*: samples were placed in a stainless-steel saucepan, covered with water and brought to a boil over high heat. Heat was reduced to maintain a simmer and samples were taken out of the water at 3 or 8 min. Following cooking, eggs were drained, rinsed with cold water, and the shell was removed. Once the shell was removed, each egg was weighed. *Frying*: a non-stick frying pan was brought to temperature over high heat, the temperature was reduced to medium, four sprays of non-stick spray oil were applied and an egg was added into the pan. The egg was turned halfway through cooking and samples were collected at 3.5 or 8 min. *Poaching*: water was brought to simmer in a stainless-steel saucepan over medium heat. Once simmering, an egg was added and cooked for 3 or 10 min. Following cooking, eggs were removed and drained well using a slotted spoon. *Scrambling*: a non-stick frying pan was heated over medium heat, four sprays of non-stick spray oil were applied and an egg was added and stirred using a stainless-steel spoon. Eggs were cooked for 50 s or 2 min.

### 2.2. Sampling

All raw and cooked samples were homogenised in 2-(*N*-cyclohexylamino)ethanesulfonic acid (CHES)/HEPES buffer, using a stand homogeniser. Cooked samples were homogenised in approximately an equal volume of buffer to egg weight and raw samples were homogenised in approximately double the volume of buffer to weight of egg. Homogenates (3 mL aliquots) were purged with N_2_ and stored at −80 °C for subsequent folate analysis.

### 2.3. Determination of Total Folate Content of Samples

All preparative and analytical procedures were performed under golden yellow light and contact with air was minimised by purging with N_2_. The total folate content of egg samples was measured in duplicate for all experiments by microbiological assay with *Lactobacillus casei* preceded by thermal extraction with tri-enzyme treatment. 

### 2.4. Thermal Extraction with Tri-Enzyme Treatment

All preparative and analytical procedures were performed as originally described by Tamura [27] and modified by McKillop et al. [26] with the following additional modifications. Solutions of α-amylase and protease (Sigma-Aldrich Company Ltd., Poole, UK) were prepared fresh each day by dissolution in ultrapure water (Purelab Prima, Davidson & Hardy Ltd., Belfast, UK) at concentrations of 50 mg/mL and 2 mg/mL, respectively. Trace levels of folate were removed from the enzyme solution by treatment with activated charcoal and filtration through a 0.2 µm syringe filter.

Thawed samples (500 µL) were mixed in 50 mL Oak Ridge centrifuge tubes (Nalgen, Rochester, NY, USA) with 5 mL CHES/HEPES buffer, pH 7.85, which had been taken to 100 °C by immersion in a boiling water-bath. Samples were boiled at 100 °C for 10 min. Following rapid cooling on ice, 500 µL α-amylase was added to each tube and incubated for 1 h at 37 °C. The enzyme was thermally deactivated, and 500 μL protease was added to each tube and incubated for 2 h at 37 °C. The enzyme was thermally deactivated and samples were centrifuged at 3000× *g* for 10 min. The resulting supernatant was divided into 3 × 1.5 mL aliquots, flushed with N_2_, and stored for up to 3 months at −80 °C until folate determination.

### 2.5. Microbiological Assay with Lactobacillus casei

Total folate content was determined by microbiological assay as previously described by Molloy and Scott [28]. Calibration of the assay was performed using FA (Sigma-Aldrich Company Ltd.) as a standard. For quality-control (QC) purposes, lyophilized pig liver (CRM 487, European Commission, Institute for Reference Materials and Measurements, Belgium) was used. The inter-assay coefficient of variation for folate content of QC samples was 7.1% (*n* = 12). Samples for all storage conditions were analyzed together and samples from each cooking method in addition to raw samples were analyzed together. All dilutions were carried out in a 0.5% sodium ascorbate solution using an automated dilutor (Hamilton, Bonaduz AG, Bonaduz, Switzerland).

### 2.6. Statistical Methods

The sample size for this study was estimated by using the results for natural folate content of enriched eggs from our previous published work [22] and considering potential losses of folate during cooking. It was estimated that a sample size of six eggs per group would be required in order to discriminate 15% to 20% difference between the cooked and the raw eggs with a power of 80% at α = 0.05.

All statistical analysis was performed using the Statistical Package for the Social Sciences (SPSS, version 15.0; SPSS UK Ltd., Chertsey, Surrey, UK). Differences in folate content between egg type, storage temperature and length of storage were compared using one-way analysis of variance (ANOVA) with the Scheffe post hoc test and independent and paired samples *t* tests. Differences in folate content between cooking methods and cooking time points were established by ANOVA with the Scheffe post hoc test. For normalization purposes, variables were log transformed as appropriate prior to statistical analysis and results were considered significant at *p* < 0.05. Results are expressed as means ± SD.

## 3. Results

### 3.1. Effect of Storage on Egg Folate Content

Table 1 presents the total folate content of eggs following storage at either refrigerator (4–7 °C) or room (18–20 °C) temperature for a period of up to 27 days. On day 1, prior to storage, the folate content of the enriched eggs was found to be significantly higher than that of the un-enriched both barn (*p* < 0.001) and free-range (*p* < 0.001) eggs. Furthermore, within the un-enriched eggs, the folate content of the free-range eggs was significantly higher compared to that of the barn eggs (*p* < 0.001). Again, on day 27, there was a significantly higher folate content of the enriched eggs as compared to the un-enriched both barn (*p* < 0.001) and free-range (*p* < 0.001) eggs, with the folate content of the un-enriched free-range eggs also being significantly higher than that of the barn eggs (*p* < 0.001). No changes in the folate content were observed between the un-enriched barn and free-range eggs analyzed on day 1 and those that were stored for 27 days. There was also no significant difference in the folate content of the enriched eggs analyzed on day 1 and those analyzed at day 7, day 14, day 21 and day 27. Un-enriched both barn and free-range eggs stored at refrigerator temperature were not found to differ significantly in folate content compared with the eggs that were stored at room temperature. Moreover, at all time points, there was no significant difference in folate content in the enriched eggs stored at refrigerator temperature and those that were stored at room temperature.

### 3.2. Effect of Cooking on Egg Folate Content

Table 2 presents the total folate content of enriched eggs following four typical cooking procedures. No statistically significant difference in folate content was observed between the raw eggs and eggs subjected to boiling, frying, poaching and scrambling. For all cooking methods, it was found that cooking time did not significantly affect folate content. Compared to raw values, no significant difference in folate content was observed in eggs that had been cooked for the minimum time and those that had been cooked for the maximum time.

## 4. Discussion

The purpose of this study was to investigate the effect of storage and typical cooking methods on the stability of natural folates in folate-enriched eggs. The results showed that the folate content was not adversely affected by storage at either refrigerator or room temperature as used by retail outlets and consumers. Moreover, folate in enriched eggs remained stable when cooked by four typical cooking methods, including boiling, frying, poaching and scrambling, even after cooking for prolonged time periods.

Unlike the synthetic vitamin form, FA, natural food folates are chemically unstable [29] and therefore can be prone to losses during storage, processing and cooking [24,25,26]. The current results however showed that neither the temperature (refrigerator or room), nor the duration of storage for up to 27 days (i.e., from date of laying to Best Before date as applied by producers), resulted in significant folate loss from eggs, whether they were un-enriched or folate-enriched. Similarly, previous reports showed only minimal folate losses during storage for beef liver and a variety of fruits and vegetables [30,31,32,33]. Although there is limited evidence specifically relating to the stability of folate in eggs, one previous report—in agreement with the current study—showed that folates remained stable when eggs were stored for 28 days at 4 °C [23].

Of perhaps greater note, the current results showed that cooking of folate-enriched eggs had no significant effect on folate stability, regardless of the method used. Folate in egg yolk from conventional eggs was previously shown to be fairly stable following boiling for 15 min, with folate retention estimated to be 73%–94% [34,35]. The current results on folate-enriched eggs showed even greater stability of the natural folate content (with retention rates of almost 100%), after boiling, frying, poaching or scrambling, even for prolonged time periods (corresponding to ‘hard’ consistency). In contrast, much older studies reported substantial losses of folates, of up to 70%, from eggs which were boiled, fried, poached or scrambled [36]. This discrepancy is most likely the result of improvements in the methodology for food folate analysis, which has advanced considerably in recent decades, particularly with respect to the extraction buffer. The current study used 2-mercaptoethanol as well as ascorbic acid, whereas older studies used ascorbic acid only in the preparation of the buffer used to extract folates from foods prior to analysis by microbiological assay [36]. The additional use of 2-mercaptoethanol as an antioxidant to protect folates during sample processing analysis was previously reported to achieve greater folate recoveries [37,38]. 

In contrast to the current findings, showing that boiling even for the maximum cooking time of 8 min resulted in no significant loss of natural folates from eggs, previous research has generally found that processes involving direct contact between the food product and cooking liquid, such as blanching and boiling, can lead to leaching of the vitamin. Thus, considerable losses of folate have been reported with boiling or blanching of green vegetables and legumes, both under domestic conditions and on an industrial scale [24,25,26,30,39,40]. Longer cooking times have been reported to result in even more substantial folate losses [30,32,41]. The protective presence of the egg shell during boiling is unlikely to be the explanation for such inconsistencies, since poaching eggs (thus subjecting the egg to direct contact with the cooking water) also resulted in no significant loss of folate in the current study. It is possible that poaching, by causing the proteins in the egg white to coagulate, may provide a protective barrier around the yolk where the majority (99%) of egg folate is located [42]. Another possible explanation for the greater folate stability in egg during boiling may be attributable to its antioxidant content and particularly the amino acid cysteine, which is abundant in eggs and may result in greater folate retention compared to foods deficient in these substances [35]. Likewise, previous reports also showed that folate losses were minimal after boiling, frying or grilling of various animal foods (beef, fish, poultry and liver) known to have a high content of cysteine [26,33,43].

The current findings, that folates in enriched eggs remain stable with storage and cooking, may have important implications for human health. The consumption of one folate-enriched egg per day (containing 75 μg folate per egg) would thus provide an effective means of increasing dietary folate intakes in consumers, thus improving folate status and potentially, the related health benefits. Given that health promotion strategies for increasing folate status based on FA supplements have proven to be ineffective at reducing the occurrence of NTDs in Europe [11], and mandatory FA fortification policies are controversial [10], alternative approaches for optimizing folate status should be considered. Regular consumption of folate-enriched eggs would enhance folate intake without the perceived safety concerns associated with FA fortification. These novel eggs would be particularly beneficial in countries without a mandatory FA fortification policy which ensures more optimal folate intakes on a population-wide basis. As eggs are generally not consumed raw, the current findings demonstrate that consumers would be able to cook folate-enriched eggs by their preferred method, following storage for periods up to the Best Before date, with no risk of folate losses. Although further research is necessary to demonstrate the effectiveness of folate-enriched eggs in increasing biomarker status of folate in consumers, our previous study suggests that egg folate will prove to be highly bioavailable [44].

We previously successfully described the development of novel folate-enriched eggs through the addition of supplemental FA to the hen’s diet, increasing the folate content by 2.5-fold, from 32 μg to 75 μg per average sized egg (i.e., 50 to 130 μg/100 g) [22]. This approach relies on the hen’s ability to convert high amounts of FA added to the feed into the natural forms of the vitamin (predominantly 5 methyltetrahydrofolate) which then transfer into the egg, with the final product containing a minimal amount (less than 10%) of unmetabolized FA [22]. In the present study, the folate content of folate-enriched eggs was shown to be significantly higher than unenriched eggs, either free-range or barn eggs, by 1.9-fold and 3-fold, respectively. The greater extent of enrichment (via FA supplementation of the hen’s diet) in the latter case was owing to a lower folate content of the unenriched product, with our results showing that the folate content of unenriched free-range eggs was significantly higher than unenriched barn eggs. The finding that conventional (i.e., unenriched) free-range eggs have higher folate content than barn eggs, as observed here for the first time, may possibly be explained by the fact that free-range hens have access to fresh forage which could provide an additional folate source to their daily feed, whereas barn hens are fed only with processed feed of minimal folate content.

The main strength of the study is that the individual experiments were carefully designed to investigate the stability of folates in eggs under the conditions of storage and cooking typically used by retailers and consumers. Prior to the cooking experiments, we carried out preliminary testing for each cooking method in order to identify the specific time required to achieve the ‘soft’ and ‘hard’ consistency of eggs, in order to ensure that our results would be directly applicable to consumer practices. In addition, we applied robust analytical methodology (based on *Lactobacillus casei* microbiological assay following thermal extraction and tri-enzyme treatment) to determine the folate content of eggs, and all necessary measures were taken to preserve natural folates during the laboratory analysis, providing confidence in the validity of our results. One limitation is that we investigated the stability of folate-enriched eggs as a food but not when used as an ingredient in composite meals and bakery products. However, given the consistency of our results showing that folates in enriched eggs are highly stable, we anticipate minimal or no folate losses if enriched eggs were used in meals or bakery products rather than consumed as foods. 

## 5. Conclusions

The current findings demonstrate that natural folates in novel folate-enriched eggs remain highly stable with little or no losses, following storage at refrigerator and room temperature for periods up to the Best Before storage time of 27 days, or when cooked by boiling, frying, poaching and scrambling, even for prolonged periods. These results are particularly relevant given that mandatory FA fortification remains non-existent in the UK and other countries in Europe, resulting in the widespread sub-optimal folate status of populations. The consumption of these novel folate-enriched eggs would offer consumers one alternative and cost efficient means of increasing folate intake and potentially protecting against disease. Current egg consumption of British women is estimated at 3–4 eggs/week [45], however evidence shows that egg consumption can be safely increased to 14 per week, with no unfavorable effect on plasma LDL cholesterol [46]; thus making folate-enriched eggs a potentially viable contributor to the folate intake of consumers. Further research, in the form of human intervention studies, is necessary to demonstrate the effectiveness of folate-enriched eggs in enhancing the biomarker status of folate in consumers.

## Figures and Tables

**Table 1:** Total folate content (μg/100 g) of eggs following 27 days storage at refrigerator temperature (4–7 °C) or room temperature (18–20 °C) ^1^.

	Folate Concentration (μg/100 g)						
	Un-Enriched Barn		Un-Enriched Free-Range		Folate-Enriched ^2^		*p*-Value ^3^
	4–7 °C	18–20 °C	4–7 °C	18–20 °C	4–7 °C	18–20 °C	
Day 1	41.4 (2.8) ^a^		65.6 (18.5) ^b^		123.2 (12.4) ^c^		<0.001
Day 7	N/A		N/A		107.9 (9.6)	127.0 (26.9)	0.17
Day 14	N/A		N/A		134.0 (16.9)	115.1 (24.3)	0.21
Day 21	N/A		N/A		119.5 (19.7)	108.2 (14.7)	0.28
Day 27	36.6 (11.3) ^a^	43.9 (5.9) ^a^	70.0 (16.4) ^b^	65.9 (7.2) ^b^	122.0 (27.7) ^c^	123.7 (18.3) ^c^	<0.001

^1^ Values are presented as mean (standard deviation); *n* = 6 for each type of eggs at each time point; ^2^ Folate enriched eggs were free-range; ^3^ Differences in folate content between egg type, storage temperature and length of storage were compared using analysis of variance (ANOVA) with the Scheffe post hoc test and independent and paired samples *t* tests. Different letters denote differences between groups (*p* < 0.001). N/A = Not applicable (not sampled/analyzed).

**Table 2:** Total folate content (μg/100 g) of folate-enriched eggs following four typical cooking treatments ^1^.

Cooking Method	Folate Concentration (μg/100 g)			*p*-Value ^2^
	Raw (*n* = 6)	Time 1 ^3^ (*n* = 6) ^4^	Time 2 ^3^ (*n* = 6) ^4^	
Boiled	135.7 (22.5)	125.2 (28.6)	145.4 (20.5)	0.402
Fried	135.7 (22.5)	137.2 (11.7)	139.1 (12.1)	0.730
Scrambled	135.7 (22.5)	133.9 (23.0)	132.5 (35.1)	0.616
Poached	135.7 (22.5)	126.9 (10.8)	132.7 (19.1)	0.597

^1^ Values are presented as mean (standard deviation); ^2^ Differences in folate content between cooking methods and cooking time points were compared using analysis of variance (ANOVA) with the Scheffe post hoc test; ^3^ Time 1 and time 2 refer to sampling times and generally correspond with the ‘soft’ and ‘hard’ consistency of the cooked egg, respectively. Time 1 and time 2 differed according to cooking method: for Boiled, 3 and 8 min; for Fried, 3.5 and 8 min; for Scrambled, 50 s and 2 min; for Poached, 3 and 10 min. Times were determined from preliminary cooking experiments (see text); ^4^ For Boiled, at both Time 1 and Time 2, *n* = 5.
